# Proposal for a Security Management in Cloud Computing for Health Care

**DOI:** 10.1155/2014/146970

**Published:** 2014-02-19

**Authors:** Knut Haufe, Srdan Dzombeta, Knud Brandis

**Affiliations:** Persicon Corporation, Friedrichstraße 100, 10117 Berlin, Germany

## Abstract

Cloud computing is actually one of the most popular themes of information systems research. Considering the nature of the processed information especially health care organizations need to assess and treat specific risks according to cloud computing in their information security management system. Therefore, in this paper we propose a framework that includes the most important security processes regarding cloud computing in the health care sector. Starting with a framework of general information security management processes derived from standards of the ISO 27000 family the most important information security processes for health care organizations using cloud computing will be identified considering the main risks regarding cloud computing and the type of information processed. The identified processes will help a health care organization using cloud computing to focus on the most important ISMS processes and establish and operate them at an appropriate level of maturity considering limited resources.

## 1. Introduction

A fundamental step for the success of tapping health care into the cloud is the in-depth understanding and the effective enforcement of security and privacy in cloud computing [1]. Despite the potential gains achieved from the cloud computing of e-health services, the information security is still questionable and the security problem becomes more complicated under the cloud model [2].

Cloud computing as actually one of the most popular themes of information computing is still at the wish list of many organizations [3] and one of the most important current research topics [4]. Cloud computing environments provide a great opportunity to provide e-health services in different scenarios in an effective and simple way [5].

One of the most important health care changes over the past couple of decades was the growing interest in health information privacy. Security and protecting the privacy and security of health information are a continuous process [6]. Particularly the security of health information is a critical responsibility of every health care organization[7].

Given that from a security perspective necessary processes and measures need to be planned and implemented. This is especially important while outsourcing information computing services in a cloud to assure an appropriate level of information security. Actually a specific framework for security management in cloud computing for health care does not exist.

To address this open research question, in this paper we propose a framework for security management in cloud-based scenarios. The rest of this work is structured as follows: in Section 2 we assess the current state of the art in the area, while Section 3 gives an overview of the framework.  Section 4 presents results from the evaluation of the framework, while Section 5 summarizes the main findings and gives an overview of future research activities.

## 2. State of the Art

### 2.1. Cloud Computing

Cloud computing in its various models can be understood as a form of the well-known outsourcing of information computing services [8].

In the last years, cloud computing is evolved from a marketing hype to a serious alternative to classical information computing [9] or even a long-held dream of computing as a utility [4, 10]. Some are already considering cloud computing as a paradigm change in information computing [3, 11]. Nonetheless, using cloud services is an important strategic decision [12].

Basic elements of cloud computing are the delivering of scalable computing services as a combination of hard- and software in a virtual environment with a usage-bound payment [13]. Additionally the shared usage of computing resources by more than one customer is a basic element of cloud computing [14]. Cloud systems have shifted traditional on-premise software products towards new and service oriented solutions [4].

While different definitions of cloud computing exist, the US National Institute of Standards and Technology [14] categorized cloud computing service models as follows:

- Infrastructure as a Service (IaaS),
- Platform as a Service (PaaS),
- Software as a Service (SaaS),

and cloud computing deployment models as follows:

- private,
- community,
- public,
- hybrid.

Regarding [10] new in cloud computing are

- the illusion of infinite computing resources available on demand,
- the elimination of an upfront commitment by Cloud users,
- the ability to pay for use of computing resources on a short-term basis as needed.

Of specific relevance are works in the area of the governance of cloud computing offerings; see [15] for an overview.

### 2.2. Security Management

In the last years, the continuous increasing dependency of nearly all organizations on appropriate secure information processing was stated practically, in relevant standards and frameworks as well as in the literature, for example [16, 17] and [18, page 5].

Standards for the management of information security and collections of best practice measures were developed and established [18–21]. Important standards for the development and operation of an information security management system (hereinafter referred to as “ISMS”) are the ISO 270xx as well as the standards of the German Federal Office for Information Security (hereinafter referred to as “BSI”) and the IT Grundschutz catalogues of the BSI in the German-speaking countries. Core principle of each ISMS standard is the well-known plan-do-check-act cycle [18, 19] which is used to structure ISMS processes.

### 2.3. Security Management in Cloud Computing

Security, in particular, is one of the most argued-about issues in the cloud computing field and the cornerstone of cloud adoption [4]; several enterprises look at cloud computing warily due to projected security risks [22] and security issues have prevented businesses from fully accepting cloud platforms [4]. Research regarding the integration of security in cloud computing is still necessary [23]. Managing security across an enterprise is one of the many business problems that organizations must solve in order to accomplish their missions. An organization's security strategy and goals must be framed in the context of risk [24]. So the specific risks according to cloud computing need to be assessed and treated in the risk management process. Specific security and privacy risks regarding cloud computing, respectively, arise from the following:

- authentication and access control [25] include physical access issues as well as identity and credential management [4],
- shared usage of computing resources (except private clouds if managed by the organization itself)—data in the cloud typically resides in a shared environment, but the data owner should have full control over who has the right to use the data and what they are allowed to do with it once they gain access [25],
- virtualization has become an indispensable ingredient for almost every cloud [26] and comes with several risks [27],
- outsourced and distributed computing (except private clouds if managed by the organization itself)—depending on the IT outsourcing risk appropriate risk treatment measures need to be developed [28],
- mobile access/access via internet—it is popular to access the cloud storage by mobile devices; this application suffers data security risk, especially the data leakage and privacy violation problem [4, 29],
- flexible and rapidly changeable services and service providers—the old advice “never touch a running system” cannot be followed anymore in cloud environments built with the intention to enable fast change [30].

In the health care sector, the general cloud computing risks are concretized as the following [31].

- Availability: as most of the health care providers would be using e-health cloud services, so to work continuously and effectively, services and data should be available all the time without performance degradation.
- Reliability: using cloud computing for such a sensitive field requires reliability for the provided services.
- Data management: a good database management is required for handling such diversified data.
- Scalability: e-health cloud would be having hundreds of health care providers with millions of patients.
- Flexibility: different health care providers might be having different requirements.
- Interoperability: as there are multiple cloud service providers, services of e-health cloud for a client could be provided by different service providers; therefore they all should work on same framework.
- Security: as many service providers could provide the e-health cloud services, and it would be used by many health care providers, therefore their security risk would be very high. When a single health care provider is using its own IT infrastructure then it will not be problem of security as it could monitor its network effectively but on a shared network various authentication methods and access controls would be required.
- Privacy: amongst all the issues of e-health cloud, the most important one is privacy.
- Organizational change: if e-health cloud is used in a health care organization, then many changes would be done like new policies, procedures, and workflows as well changes in the process of how documentation is done.
- Data ownership: in health care sector still there is no clear guideline for ownership of patient's record.
- Privacy, trust, and liability issues: as cloud is on Internet, there is a risk of data leakage, private data exposure, and data loss which could result in loss of reputation of health care provider as well as patient's trust.
- Usability and end users experiences: e-health cloud success lies in the fact that it is adopted by patients, health care professionals, management, and insurance companies.

Those risks and their consequences need to be analyzed in depth and considered while planning for the usage of cloud services for health care, defining necessary security measures, and using cloud services.

For this a detailed individual risk assessment needs to be performed [12].

## 3. The Framework

Organizations need to identify and manage many activities in order to function effectively and efficiently. Any activity using resources needs to be managed to enable the transformation of inputs into outputs using a set of interrelated or interacting activities—this is also known as a process [32, page 8]. In other words, a process is a set of interrelated or interacting activities which transforms inputs into outputs [33].

This section describes the proposed process framework designed to guide information security efforts in general as well as an identification of core ISMS processes for cloud computing in health care.

### 3.1. General Framework

The initial and most high-level process regarding ISMS is described in ISO 27003 as an ISMS planning process [34, page 2]:

- obtaining management approval for initiating an ISMS,
- defining ISMS scope and ISMS policy,
- conducting organization analysis,
- conducting risk assessment and risk treatment planning,
- designing the ISMS.

According to ISO 27000/27001, ISMS processes, which need to be designed, are

- *information security risk assessment process* [19, page 3] which is an overall process of risk analysis and risk evaluation [32, page 5],
- *information security risk treatment process* [19, page 4] which is a process to select and implement measures to modify risk [32, page 5]; controls are now determined during the process of risk treatment, rather than being selected from Annex A of ISO 27001 [35, page 4],
- *resource management process*, which ensures that necessary resources are determined and provided [19, page 5],
- *processes to assure necessary awareness and competence* [19, page 5], where the process of creating awareness may be regarded as a form of communication [35, page 12],
- *communication processes* [19, page 6], including internal and external communication as well as marketing for the ISMS,
- *documentation control process* [19, pages 6, 7],
- *requirements management process* [19, page 7],
- *change management process* [19, page 7],
- *process to control outsourced processes* [19, page 7],
- *performance evaluation process* [19, pages 7, 8], containing *monitoring* (the performance of ISMS needs to be monitored in terms of verification and reporting of security control implementation), *measurement* (a measurement system used to evaluate performance in information security management and feedback suggestions for improvement needs to be established [32, page 11]), analysis, and evaluation,
- *internal audit process* in terms of planning and conducting internal audits as part of an audit program [19, page 8],
- *management review process* [19, page 8],
- *improvement process* [19, page 9],
- *information security incident management process* [32, page 11].

**Figure 1:**
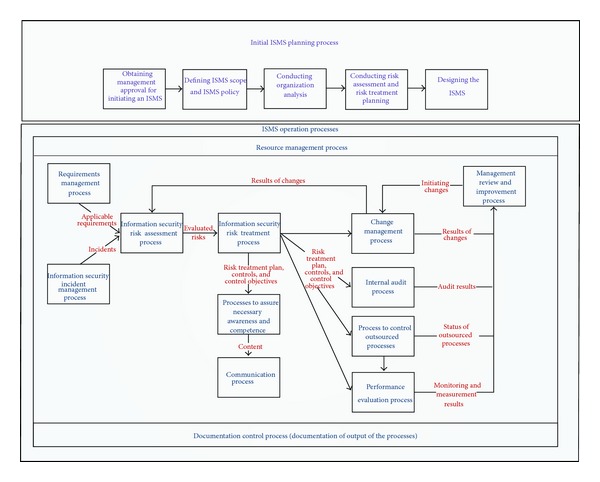
ISMS process framework.

Figure 1 shows the ISMS processes and the interaction at a high-level basis. The process begins with a requirements management process which provides relevant requirements as an input for the information security risk assessment process. Another process which provides continuously input for the assessment of risks is the information security incident management process. Results of the risk assessment process are evaluated risks which are needed in the risk treatment process. Results of the risk treatment process are a risk treatment plan, controls, and control objectives. Those results are used in various processes to

- assure an appropriate awareness and competence using appropriate communication,
- regularly check the appropriateness of the chosen controls and control objectives in the internal audit process,
- control outsourced processes,
- evaluate the performance of the controls and the ISMS in general.

Another core process in which the risk treatment plan, controls, and control objectives are used as an input is the change management process. The change management process also delivers results of changes to the risk assessment process to include them in the assessment of risks.

Results of changes, internal audits, the status of outsourced processes, and monitoring and evaluation results are reviewed within the management review and improvement process which initiates changes.

Supporting processes are the resource management process and the documentation control process.

### 3.2. Health Care Framework

While managing information security of health care organizations which are using cloud computing needs to consider more beside the core processes “*risk assessment*” and “*risk treatment*.” Based on our experience with such organizations the following ISMS processes seem to be particularly important.

*Requirements Management Process*. Particularly for health care an appropriate protection of personal data needs to be ensured considering specific legal and compliance requirements like national data protection laws and health care specific requirements. Also questions like “who owns the data?” should be answered in this context [36].

*Process to Control Outsourced Processes*. Given that cloud computing in its various models is a form of outsourcing of information computing services, the process to control those outsourced processes is key to information security. As known from classic outsourcing the compliance of the service provider with the defined requirements should also be audited frequently while using cloud services. An increased usage of different and changing subservice providers (chaining) is often used to increase the flexibility of the service provision to keep it always in line with the demand. As a result of this the changing subservice providers and services as well as the location of the computing are mostly nontransparent for the customer. The involved (sub)service providers, locations, and countries in which the information computing is performed as well as specific security requirements and measures should be defined in the contract between health care organization and cloud service provider.

*Information Security Incident Management Process*. For all significant or informative incidents, basic data (what, who, when, where, risk, and consequences) should be logged so that it can be passed on to the relevant people (notify), so that they can recommend and/or take the necessary local action [37]. Considering many involved parties using cloud computing incident management processes for all involved parties and their interfaces should be defined to ensure appropriate information of the health care organization using cloud computing of relevant incidents.

Also for biomedical researchers, those processes are important because their work includes image analysis, data mining, protein folding, and gene sequencing which requires computing capacity as well as an appropriate management of information security [38].

## 4. Evaluation Results

Evaluation of the proposed framework is currently being conducted in a real-life setting within two organizations.

**Figure 2:**
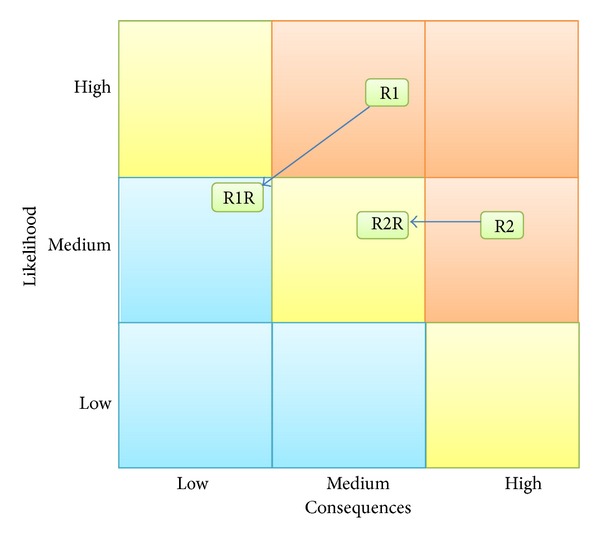
Risk map.

Organization 1 is a pharmaceutical company. Currently, the framework is being implemented in the whole organization. The implementation led to a documented and evaluated set of requirements of risks where previously only a subjective and not documented understanding of requirements and risks existed. Particularly, in two cases this helped the organization to focus on resources to treat major risks which were previously overlooked. Additionally, two audits of outsourced processes—software development and data center housing—were performed in which additional risks and necessary improvements were identified.  Figure 2 shows an anonymized example of the resulting risk map with two risks regarding the outsourced processes (R1 and R2) as well as the resulting risk after risk treatment (R1R and R2R).

**Figure 3:**
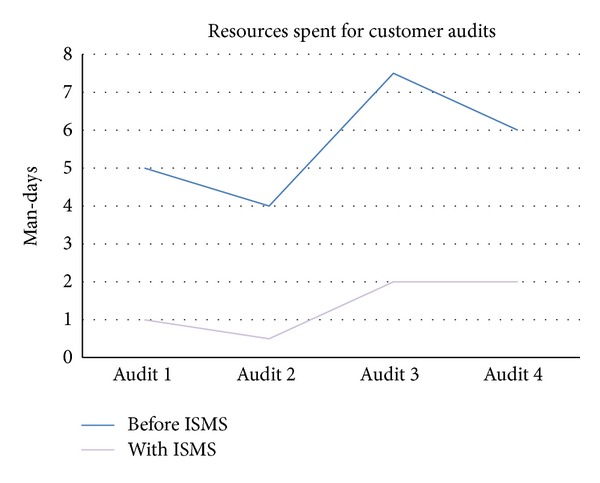
Resources needed to conduct customer audits.

Organization 2 is an IT service provider which also provides services to health care companies. Currently, the framework is being implemented in the whole organization. Key benefit is also a broad understanding of requirements of the different customers and related risks from an information security perspective and the resulting precise usage of limited resources. Especially the documentation of the results of requirements analysis, risk assessment and treatment enables the organization to deal with audits of their customers more efficient as shown in Figure 3. Resources to search and provide relevant information within customer audits and within the change management process decreased after the initial implementation of the ISMS by an average of 70%.

Preliminary results from this evaluation seem to confirm the applicability of the proposed framework to address the posited objectives.

In both cases especially an appropriate identification and understanding of relevant requirements were key to provide significant benefits. All the following processes rely on a proper understanding of the relevant requirements. Missing or wrong information regarding relevant requirements results in uncertain or wrong decisions and finally in higher cost and/or risks compared with decisions while all relevant requirements were identified and understood.

So cloud computing combined with identified and understood security requirements also enables tailored on demand security controls as a benefit [4].

## 5. Conclusions and Future Work

Research on the various security issues regarding information computing in health care environments has been done in the past. While other researchers focus on specific issues like access control or cryptographic controls [1, 36] a broader view at the ISMS processes is also required.

As shown in this work from the perspective of a health care organization using cloud computing the processes

- information security risk assessment,
- information security risk treatment,
- the control of outsourced processes,
- requirements management,
- information security incident management

are key to ensuring an appropriate information security.

Considering this result and limited resources as well as ensuring an efficient use of those resources, not every ISMS process should be established and operated at the same level of maturity.

Therefore a health care organization using cloud computing should focus on the identified processes of information security risk assessment, information security risk treatment, the control of outsourced processes, and requirements management. Particularly for these processes an adequate level of maturity is needed.

In this context future, work is necessary to develop a more detailed framework of ISMS processes (input, output, and interfaces) and their interaction at an activity level to ensure an appropriate interaction of the ISMS processes.

While not every ISMS process needs the same level of maturity, also an approach should be developed to identify the appropriate level of maturity using a proper maturity level model.

By considering a maturity level model for ISMS processes combined with an approach for the determination of the necessary maturity level, the appropriateness of an ISMS can be made transparent and unnecessary costs of information governance can be avoided.
